# Effect of an early time restricted eating Mediterranean diet compared to naltrexone/bupropion on liver fibrosis in people with cardiometabolic risk factors in a hospital outpatient clinic: study protocol for a randomised controlled trial (MEDFAST trial)

**DOI:** 10.1136/bmjopen-2025-113058

**Published:** 2026-06-25

**Authors:** Carmen A W Dietvorst, Laura A M Konings, Alina N Saidi, Tessa Noordermeer, Martien van Wenum, Robin P Peeters, Kirsten A Berk, Manuel Castro Cabezas

**Affiliations:** 1 Department of Internal Medicine, Division of Dietetics, Erasmus MC, Rotterdam, The Netherlands; 2 Department of Internal Medicine, Franciscus Hospital, Rotterdam, The Netherlands; 3 Department of Internal Medicine, Erasmus MC, Rotterdam, The Netherlands; 4 Department of Clinical Chemistry, Franciscus Hospital, Rotterdam, The Netherlands; 5 Department of Gastroenterology and Hepatology, Franciscus Hospital, Rotterdam, The Netherlands; 6 Julius Clinical BV, Zeist, The Netherlands

**Keywords:** Diabetes Mellitus, Type 2, NUTRITION & DIETETICS, Obesity, Overweight, Cardiovascular Disease, Hepatology

## Abstract

**Introduction:**

Metabolic dysfunction-associated steatotic liver disease (MASLD) is a progressive condition that may lead to liver cirrhosis and hepatocellular carcinoma. It is strongly associated with cardiometabolic diseases such as type 2 diabetes (T2D) and obesity. Weight loss is a key therapeutic strategy to treat MASLD. In recent years, there has been increasing attention to weight-reducing medication, but promising dietary interventions—such as early time restricted eating (eTRE)—have also been developed. Whether these dietary interventions are a good alternative to weight-reducing medication remains unclear, as direct comparative studies are still lacking. Therefore, this study will compare the effects of an early time restricted eating Mediterranean diet (eTRE-MD) intervention with naltrexone/bupropion (N/B) treatment on liver fibrosis, measured as liver stiffness (LSM) by transient elastography, in individuals with cardiometabolic risk factors.

**Methods and analysis:**

We will conduct a randomised controlled trial with a planned sample size of 70 overweight (Body mass index, BMI >27 kg/m2) adults aged 18–75 years with T2D, hypertension, dyslipidaemia or obesity and moderate to severe liver fibrosis, measured as LSM (>7.0 kPa and <13.6 kPa). The diet group will receive eTRE-MD, with a 10-hour eating time restriction (8AM–6PM). The medication group will receive 32 mg/360 mg of N/B (after a dose increase period following Summary of Product Characteristics). The main study endpoint is the between-group difference in liver fibrosis (kPa) from baseline to 6 months. Secondary endpoints are liver steatosis, weight, body composition, cardiovascular risk factors, quality of life, patient satisfaction and compliance. General linear models for repeated measurements will be applied for statistical analysis of the data.

**Ethics and dissemination:**

Ethical approval from the Medical Research Ethics Committees United (MEC-U) has been obtained. The results of the study will be submitted for publication in a peer-reviewed journal.

**Trial registration number:**

NCT06845345.

**Strengths and limitations of this study**

- This study uses a randomised controlled design to compare two active interventions.
- The planned sample size provides adequate statistical power to detect between-group differences.
- Blinding is not feasible because the interventions differ substantially, but the primary outcome is an objective clinical measure with low risk of bias.
- Liver steatosis and fibrosis are assessed using transient elastography rather than liver biopsy, which is the diagnostic gold standard.

## Introduction

 Metabolic dysfunction-associated steatotic liver disease (MASLD) is a progressive disease ranging from liver steatosis to more severe metabolic dysfunction-associated steatohepatitis (MASH). This involves damage to hepatocytes, resulting in a healing process in which scar tissue (fibrosis) is formed. In severe cases, fibrosis can lead to cirrhosis, hepatic failure or even hepatocellular carcinoma. Recent data suggest that MASLD may be present in approximately 30% of the adult world population.^1 2^ However, the prevalence of MASLD in high-risk patients such as individuals with type 2 diabetes (T2D) or dyslipidaemia can be as high as 50%.^3 4^ Besides the risks of serious liver disease, people with MASLD have a higher cardiovascular risk.^5 6^

Lifestyle modification–including weight loss, dietary changes, physical exercise and discouraging alcohol consumption–is currently the primary recommended treatment for MASLD according to the latest guidelines from the European Association for the Study of the Liver (EASL).^7^ Most guidelines highlight the Mediterranean diet as an effective dietary intervention for MASLD. A recent meta-analysis supports this recommendation, showing that the diet significantly reduces liver fat and insulin resistance in individuals with MASLD, independent of weight loss or amount of visceral fat.^8^ Furthermore, long-term adherence to the Mediterranean diet combined with carbohydrate restriction appears more effective in reducing liver fat than a fat restricted diet alone.^9^ In addition to the Mediterranean diet, other nutritional strategies have shown promise. Caloric restriction through intermittent fasting may benefit individuals with MASLD.^10^ Observational studies suggest that shifting food intake to earlier in the day is associated with a lower risk of fatty liver disease, and that intermittent fasting improves hepatic outcomes in people with T2D.^10 11^ Intermittent fasting is an umbrella term that encompasses a range of dietary interventions aimed at extending the fasting period. Among these interventions, eTRE—which limits the daily eating window to less than 10 hours, ending in the late afternoon—seems to be the most promising strategy. In a 2-week proof-of-principle controlled eating study, eTRE led to significant improvements in metabolic health, including glycaemic markers, among overweight prediabetic men, independent of changes in body weight.^12^ Additional evidence suggests that eTRE enhances insulin sensitivity and glycaemic control in individuals with T2D and promotes weight reduction in people with obesity.^13^,^16^ Given that weight loss remains a cornerstone in the management of MASLD, these findings are clinically relevant. Histological data indicate that a 3%–5% reduction in body weight can improve liver steatosis, while losses exceeding 10% may lead to regression of steatohepatitis and fibrosis.^17^ Despite these promising outcomes, only a limited number of randomised controlled trials have evaluated the effects of eTRE in individuals with MASLD. Current evidence supports its efficacy in reducing intrahepatic fat; however, its impact on liver fibrosis has not yet been clarified.^18 19^ A recent 12-week intervention combining eTRE with a hypocaloric Mediterranean diet demonstrated a significant reduction in liver fibrosis, though the short duration limits conclusions regarding long-term effects.^20^ Therefore, further long-term research is needed, combining eTRE with a Mediterranean diet to investigate the most potential dietary strategy for people with MASLD.

Besides dietary strategies, pharmaceutical interventions are increasingly being explored as effective options for the treatment of MASLD. Recently, resmetirom was approved by both the US Food and Drug Administration (FDA) and the European Medicines Agency (EMA) as the first pharmacological agent specifically indicated for the treatment of liver fibrosis in people with MASH.^21 22^ Other promising agents include glucagon-like peptide 1 (GLP-1) receptor agonists such as semaglutide, originally developed for obesity and cardiovascular risk reduction, which has now also been approved for the treatment of MASH in adults with moderate to advanced liver fibrosis.^23^ In parallel, several pharmacological treatments are already available to support weight loss, which remains a cornerstone in MASLD management. Currently approved medications for obesity in adults include orlistat, liraglutide, semaglutide and naltrexone/bupropion (N/B).^24^ Although all four have demonstrated efficacy in reducing body weight, N/B may be considered favourable due to its oral administration and relatively lower cost. N/B reduces hunger and enhances satiety, ultimately promoting weight loss. A long-term study showed that patients treated with N/B lost significantly more weight compared with placebo, and those achieving ≥5% wt loss after 16 weeks were more likely to maintain this reduction after 1 year.^25^ Despite these benefits, side effects such as nausea, constipation, vomiting, diarrhoea and dry mouth may occur. Importantly, agents such as semaglutide and resmetirom have direct cardiometabolic and liver-specific effects beyond weight reduction, making it difficult to attribute changes in liver fibrosis to weight loss alone. In contrast, N/B induces weight loss without known hepatic benefits, making it an appropriate comparator for evaluating weight-loss driven effects on MASLD, which is the aim of the current study.

To the best of our knowledge, the effectiveness of dietary intervention compared with weight loss medication has not been evaluated in people with MASLD, as pointed out in the most recent EASL guideline for MASLD.^7^ Because both lifestyle modification and pharmacological therapy are considered primary treatment strategies—and individual patients may respond differently to each—direct comparison of these approaches may help clarify their relative effectiveness and inform clinical decision-making. This study aims to compare the pharmacological treatment with N/B with a dietary intervention combining eTRE with a Mediterranean diet (eTRE-MD). We hypothesise that the eTRE-MD intervention will be more effective than N/B in reducing liver fibrosis in individuals with MASLD.

### Objectives

The primary objective of this study is to determine the difference in effectiveness of an eTRE-MD compared with naltrexone/bupropion (N/B) during 6 months on liver fibrosis, measured as liver stiffness (LSM) by transient elastography, in individuals with one or more cardiometabolic risk factors and overweight. Given that liver fibrosis is the strongest prognostic marker for adverse outcomes in MASLD, LSM is the primary objective. This study intentionally targets a more advanced MASLD population, as there is limited evidence on the effects of dietary interventions in individuals with established fibrosis. Steatosis will also be analysed to enable comprehensive phenotyping and to examine its relationship with changes in LSM and metabolic outcomes.

The secondary objectives of this study are:

1. To compare the effect of eTRE-MD vs N/B on liver steatosis during 6 months in individuals with one or more cardiometabolic risk factors and overweight.
2. To determine the difference in weight loss and body composition between eTRE-MD and N/B during 6 months.
3. To determine the difference in cardiovascular risk factors and biomarkers associated with MASLD between eTRE-MD and N/B during 6 months.
4. To determine the difference in lifestyle factors between eTRE-MD and N/B during 6 months.
5. To evaluate the difference in quality of life, treatment satisfaction, compliance and adherence between eTRE-MD and N/B during 6 months.

## Methods and analysis

### Patient and public involvement

Patient organisations were involved in the development of this study. The current research proposal has been submitted to the Dutch Diabetes Association (DVN), the Dutch Liver Patients’ Association (NLV) and the European Liver Patients’ Association (ELPA) for comment and support. They have provided feedback on the proposal, taking the patients’ interests into account.

### Trial design

**Figure 1:**
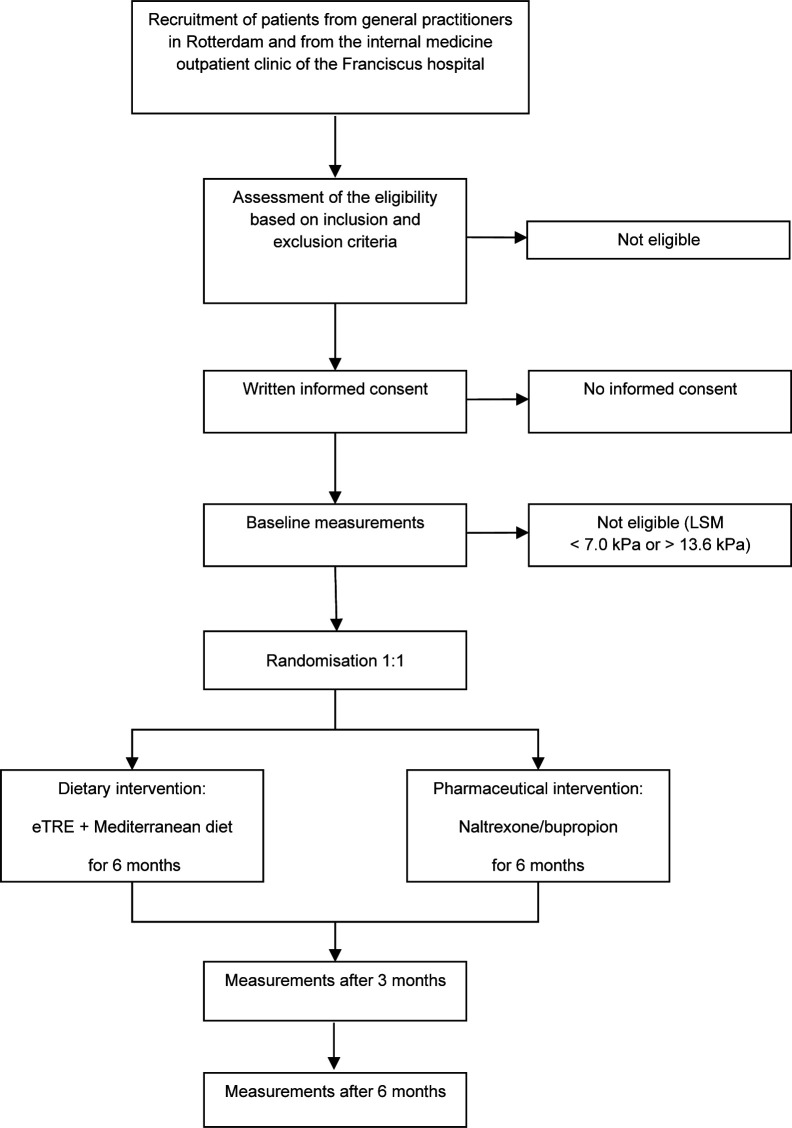
Flowchart of the trial. eTRE, early time restricted eating; LSM, liver stiffness measurement.

This study is a randomised controlled trial with a duration of 6 months. After signing informed consent, eligible participants will be randomised to eTRE-MD or N/B. Details of the informed consent form are presented in online supplemental file 1. Outcome parameters will be measured at baseline, 3 months and 6 months. Figure 1 shows the flowchart of this study.

### Trial setting

People with obesity or overweight and T2D, hypertension or dyslipidaemia, and moderate to severe liver fibrosis will be recruited from the outpatient clinic of the Franciscus hospital in Rotterdam and general practitioners in Rotterdam, The Netherlands. The study started in April 2025 and is expected to be completed in July 2027.

### Eligibility criteria

Inclusion criteria are:

- Body mass index (BMI) >30 kg/m2 or BMI >27 kg/m2 and at least one cardiometabolic risk factor (T2D, hypertension, dyslipidaemia).
- Moderate to severe liver fibrosis, measured as LSM by transient elastography (>7.0 kPa and <13.6 kPa).
- Aged 18–75 years.
- Written informed consent,

Exclusion criteria are:

- An insufficient comprehension of the Dutch language (spoken and written).
- Pregnancy, breast-feeding or intention to become pregnant.
- Participants with an established diagnosis of liver pathology like, but not limited to:
  - Hepatitis B.
  - Hepatitis C.
  - Autoimmune hepatitis.
  - Wilson’s disease.
  - Haemochromatosis.
  - Primary biliary cholangitis.
  - Primary sclerosing cholangitis.
  - Alcoholic liver disease.
- History of liver transplant, or current placement on a liver transplant list.
- History of cirrhosis and/or hepatic decompensation, including ascites, hepatic encephalopathy or variceal bleeding.
- Participants with active HIV infection and/or treatment.
- Participants with diagnosed malignancies with or without active treatment.
- Participants with history or pre-existing renal disease (estimated Glomerular Filtration Rate (eGFR) <30 mL/min/1.73 m^2^).
- Participants with corticosteroid induced diabetes (while still using corticosteroids).
- Participants using GLP-1 agonists for less than 3 months or not yet on a stable dose.
- Participants using Monoamine Oxidase inhibitors, opioids and/or methadone (due to contraindication).
- Known or suspected excessive alcohol consumption (>30 grams/day for males or >20 grams/day for females. One drink is equivalent to 10 grams of alcohol).
- Previous or planned (during the trial period) obesity treatment with bariatric surgery. However, previous interventions that have resulted in stable weight for at least the past 3 months are allowed.
- Participants with a history or evidence of any other clinically significant condition or planned or expected procedure that in the opinion of the investigator, may compromise the patient’s safety or ability to complete the study.

### Interventions

Participants will be randomised into one of the two groups:

1. Diet group: using an eTRE-MD.
2. Medication group: using N/B.

#### Diet group

Participants in the diet group will follow with a calorie restricted Mediterranean diet, with an eating window between 8 am and 6 pm for a period of 6 months. Participants are asked to stop eating at 6 pm. This results in a daily fasting window of approximately 14 hours. During the fasting time, only calorie-free drinks will be allowed. A daily calorie restriction of 500 kcal will be applied, based on the estimated energy requirement calculated using the WHO equation for participants with a BMI ≤30 and the Harris-Benedict equation for those with a BMI >30.^26^ The diet will be based on the Mediterranean food pyramid,^27^ emphasising high-fibre foods such as fresh fruits and vegetables, unprocessed whole grains, beans, seeds, herbs and spices, while limiting processed foods, red meat, sweets and alcohol. At baseline, participants receive a detailed explanation of the dietary protocol, two sample daily meal plans, recipes and a food-exchange list. Dietary adherence is assessed at the 3-month and 6-month follow-up visits using food diaries in which participants record all foods consumed and the corresponding times. The eating window is additionally evaluated at each visit through a questionnaire assessing the average latest eating time and the number of days per week participants adhere to the fasting regimen. Participants receive guidance from a registered dietitian to support correct implementation of the intervention. Consultations take place during the in-person study visits at baseline, 3 months and 6 months. Throughout the intervention period, participants may also contact the study dietitian by telephone for ongoing support. During the entire programme, 60 min of exercise per day is advised. Physical activity will be measured for 1 week with the Activ8 Activity Tracker GEN2 at baseline and at the end of the study.

#### Medication group

The medication used in the study is N/B to ensure that the comparator reflects weight loss alone, without any additional metabolic or liver-specific pharmacological effects. Participants in the medication group will take N/B (Mysimba) two times per day at a total dose of 32 mg/360 mg N/B per day for a duration of 6 months. The dosage will be built up in the first month following the stepped care approach used in the Summary of Product Characteristics (SmPC), up to maximally tolerated doses. Compliance with N/B intake is monitored by pill counting at the 3-month and 6-month visits. Participants are asked to bring all medicine packaging to each visit, and the number of remaining tablets is recorded by the researcher. Adherence is defined a priori as taking at least two tablets per day, with dose adjustments permitted based on individual tolerance. They receive usual care, including the advice of 60 min of exercise per day and standard dietary recommendations according to the guidelines for the Dutch population.^28^ During the visits at baseline, 3 months and 6 months, medication use and usual care will be evaluated by a physician in the hospital. Participants will wear the Activ8 Activity Tracker GEN2 at baseline and at the end of the study.

### Outcomes

The main study parameter is the between-group difference in absolute change in liver fibrosis during 6 months. Liver fibrosis will be measured as LSM (kPa) by transient elastography (FibroScan).

Secondary study parameters are as follows:

- Absolute change in liver steatosis (Controlled Attenuation Parameter score, FibroScan).
- Nutritional assessment: body weight (kg, Seca 888 compact digital flat scale), height (cm), waist circumference (cm), fat mass and lean body mass measured with bioelectrical impedance analysis (kg, Bodystat 500), grip strength (kg, Jamar).
- Cardiovascular risk factors: total cholesterol, low-density lipoprotein cholesterol, high-density lipoprotein cholesterol, triglycerides, apolipoprotein B (ApoB), apolipoprotein AI (ApoAI), lipoprotein (a), Hemoglobin A1c, fasting blood glucose, fasting insulin and blood pressure, measured with routine lab procedures.
- Other laboratory measurements, measured with routine lab procedures. Among others, the following routine measurements will be done: creatinine, eGFR, alanine aminotransferase (ALAT), aspartate aminotransferase (ASAT), thrombocytes, haemoglobin (Hb), leucocytes, transferrin, ferritin, total iron binding capacity, apolipoprotein B48 (ApoB48), fibroblast growth factor 19 (FGF19) and 21 (FGF21), C-reactive protein (CRP), fibrosis-4 index score (Fib-4), thyroid-stimulating hormone (TSH), free thyroxine 4.
- Physical activity (Activ8 Activity Tracker GEN2).
- Quality of life (36-Item Short Form Health Survey Questionnaire).^29^
- Patient satisfaction in case of T2D (Diabetes Treatment Satisfaction Questionnaire).^30^
- Food intake and adherence to the dietary intervention (3-day food diary).
- Demographic variables, drug use, smoking and drinking habits, medication use and compliance to the time restriction (self-developed questionnaire).

Other study parameters are as follows:

- Shift in liver fibrosis severity (categorised in fibrosis score F0-F3).
- Side effects (self-reported).
- Number of participants that drop-out (categorised by reason for drop-out).

### Study timeline

The participant timeline is presented in table 1.

**Table 1:** Schedule of enrolment and measurements

Timepoint	Enrolment	Screening	Visit 1	Visit 2	Visit 3
	- 2 weeks	Baseline		3 months	6 months
Eligibility screen	x				
Informed consent		x			
Transient elastography		x		x	x
Length		x			
Weight		x		x	x
Waist circumference			x	x	x
Body composition			x	x	x
Grip strength			x	x	x
Blood pressure			x	x	x
Laboratory measurements			x	x	x
Physical activity			x		x
SF-36 questionnaire			x	x	x
3-day food diary			x	x	x
Questionnaire demographics, lifestyle and medication			x	x	x
Consultation with dietitian (eTRE-MD group)			x	x	x
Medication dispensing (N/B group)			x	x	x

eTRE-MD, early time restricted eating Mediterranean diet; N/B, naltrexone/bupropion; SF-36, 36-Item Short Form Health Survey.

### Sample size and recruitment

Little is known in the literature about the effect of N/B on liver fibrosis. A recent study showed a decrease in LSM from 4.67 kPa to 4.14 kPa after 6 months of N/B use.^31^ The relevance of this finding is unclear, since LSM values <7.0 kPa are considered to be not clinically relevant. Studies in which participants followed a Mediterranean diet for 6 months show varying outcomes. Two studies showed that it has an average decrease of 0.4 to 2.1 kPa in liver fibrosis.^32 33^ To calculate the effect size, we took a mean difference of the two dietary studies (1.3 kPa), the mean of the N/B group (0.5 kPa) and an SD of 1.0 kPa, which is based on expert opinion due to lack of evidence in the literature. This leads to an effect size of 0.8 kPa. In this study, we will perform a two-sided hypothesis test to assess whether there is a difference in LSM after 6 months between the eTRE-MD group and N/B group. The null hypothesis (H0) states that there is no difference in LSM between the groups, while the alternative hypothesis (H1) assumes a difference of 0.8 kPa or more. Based on an effect size of 0.8 kPa with an SD of 1.0 kPa, a significance level of 0.05 and power set to 0.80, we calculated that 29 participants per group are needed. Assuming a drop-out of 10%, inclusion will be 70 participants (35 participants per group). Unpublished data from ongoing research within our department show that about 20% of people with one or more cardiometabolic risk factors in the Netherlands have a transient elastography result >7.0 kPa. This means that to include 70 patients, we will need to screen about 350 people. It is likely that the planned number of participants can be recruited as 3000 people with cardiometabolic risk factors are seen at the outpatient clinic every year. The patient population not treated at the hospital will be referred by the general practitioner when MASLD is suspected.

### Randomisation

After signing informed consent, participants will be randomised to either the eTRE-MD group or N/B group. A 1:1 randomisation will be performed via a data management system (Castor Electronic Data Capture (EDC), Amsterdam, The Netherlands) in blocks of 2, 4 and 6. Results from the randomisation will be communicated to the participant after the baseline measurements. The resulting randomisation list will be managed by the secretary of the department of Internal Medicine in the Franciscus hospital. Participant blinding will not be done, as blinding is impossible considering this type of intervention. However, the first preliminary statistical analyses of the primary outcome will be performed by an independent statistician, blinded for the allocation.

### Data collection

The recruitment, enrolment and data collection are conducted by the investigator, who is also responsible for delivering the interventions. Before the start of the study, the investigator will hold a pre-screening by phone to explain the study and discuss the inclusion and exclusion criteria. The patient is given at least 2 weeks for reflection. If the patient agrees to participate, a screening will be scheduled at the Franciscus hospital to determine the degree of liver fibrosis using transient elastography (Fibroscan).

If the participant meets the inclusion criteria, they will sign a consent form together with the researcher during the same visit, and the baseline measurements and randomisation will be performed. After 3 months (visit 2) and 6 months (visit 3), the same measurements are performed as at baseline (visit 1) (table 1). Participants in both groups will have an appointment by phone with the researcher after 2 weeks to check whether the diet or medication is being used properly and whether complaints have occurred.

### Data management

Data will be entered into a data management system (Castor EDC, Amsterdam, The Netherlands) with a consecutive participant code number (no name and any other identifying detail). The participant identification code list, which links the code number to the participant, will be safeguarded by the secretary of the department of Internal Medicine in a ‘master file’. The key to the code is only accessible by the investigators. Study data can only be accessed by the investigator team and staff of the Health Care Inspection, as stated in the informed consent form. Data will be collected and processed in accordance with the General Data Protection Regulation (EU) 2016/679 and the Dutch Act on Implementation of the General Data Protection Regulation. All data will be kept for a maximum of 25 years after the trial has finished.

### Statistical methods

All analyses will be conducted according to the intention-to-treat and per protocol principle. To be included in the per protocol analysis, a participant is required to have at least data available for two time points (baseline and T1 or T2) and to achieve 80% or higher adherence. Participants of the eTRE-MD group are considered adherent to the dietary protocol if they restrict their daily food intake to a window of less than 10 hours—specifically between 7:30 AM and 6:30 PM—on at least 5 days per week. Additionally, they must maintain a daily caloric intake that is at least 400 calories below their estimated energy requirement. Participants of the N/B group are considered adherent to the medication protocol if they consistently take a minimum of two tablets per day, with the exact dosage adjusted according to individual tolerance levels. These adherence data will be used to classify participants for the per protocol analysis and to explore the impact of medication compliance on treatment outcomes. All other non-compliant participants will be excluded from the per protocol analysis. Normality of the data and homogeneity of variances will be tested using Shapiro-Wilks test and Levene’s test. For numerical data, we will use the mean (in case of normal distribution) and median values (in case of non-normal distribution), with respectively the SD and IQR as measures of dispersion.

The primary outcome will be defined as the absolute change in liver fibrosis during 6 months. The difference in liver fibrosis change between the eTRE-MD group and N/B group will be analysed with a linear mixed model adjusted for baseline, with independent variables treatment arm, time point (T1 and T2), and the interaction between treatment arm and time point. The estimated difference between treatment arms in the liver fibrosis change at T2 (6 months) will be calculated using the estimated marginal means. Mixed modelling can efficiently handle data with missing and unbalanced time-points. It corrects for bias when absence of data is dependent on characteristics that are present in the models (missing at random, MAR). The secondary outcomes will be analysed with a linear mixed model adjusted for baseline, with independent variables treatment arm, time point (T1 and T2), and the interaction between treatment arm and time point. The estimated difference between treatment arms will be calculated using the estimated marginal means.

To account for heterogeneity in metabolic profiles, cardiometabolic condition (type 2 diabetes, hypertension, dyslipidaemia or obesity) will be included as a covariate in all primary and secondary outcome analyses. Outcomes will be analysed using linear mixed models with treatment arm, time point (T1 and T2), and their interaction as fixed effects, adjusted for baseline values. An interaction term between treatment arm and cardiometabolic condition will be explored to assess potential differential treatment effects across subgroups. Given the limited sample size, stratified analyses will not be performed unless sufficient power is available. Participants with multiple conditions will be included in the overall model and appropriately coded for subgroup membership. Sensitivity analyses may be conducted to evaluate the robustness of findings in overlapping vs non-overlapping subgroups.

### Monitoring

An independent monitor (quality officer), appointed by the sponsor, will monitor the study data according to Good Clinical Practice (GCP). The frequency of the monitoring is related to the risk of the investigation. For at least a selection of the participants, informed consent forms will be checked. Source Data Verification will minimally be performed (checking if data from the Case Report Forms (research forms/questionnaires) match with the source data (patient status, results, etc)). The quality of data will be guaranteed by using the data management software Castor EDC. The monitor will check whether all (Serious) Adverse Events (S)AEs and Suspected Unexpected Serious Adverse Reactions are adequately reported within the timelines as required by law, the presence and correctness of the informed consent forms, the delegation log and the data storage.

### Adverse events reporting and harms

Adverse events are defined as any undesirable experience occurring to a participant during the study, whether or not considered related to the intervention. All adverse events reported spontaneously by the participant or observed by the investigator or staff will be recorded in the case report forms.

A serious adverse event is any untoward medical occurrence or effect that

- results in death;
- is life threatening (at the time of the event);
- requires hospitalisation or prolongation of existing inpatients’ hospitalisation;
- results in persistent or significant disability or incapacity;
- is a congenital anomaly or birth defect; or
- any other important medical event that did not result in any of the outcomes listed above due to medical or surgical intervention but could have been based on appropriate judgement by the investigator.

An elective hospital admission will not be considered as a serious adverse event.

### Ethics and dissemination

This study has been reviewed and approved by the Medical Research Ethics Committees United (EU CT-number 2024-519774-40-00). It will be conducted according to the principles of the Declaration of Helsinki (version October 2024, Finland) and in accordance with the Medical Research Involving Human Subjects Act (WMO). Any future amendments will be shared with the research group. All participants will provide written informed consent. The trial is registered at Clinicaltrials.gov, NCT06845345.

The results of the study will be presented at scientific meetings and published in a peer-reviewed scientific journal. Summaries will be provided to the participants after publication of the manuscript.

## Supplementary material

10.1136/bmjopen-2025-113058 online supplemental file 1
