# Supplementary material for: Effect of an early time restricted eating Mediterranean diet compared to naltrexone/bupropion on liver fibrosis in people with cardiometabolic risk factors in a hospital outpatient clinic: study protocol for a randomised controlled trial (MEDFAST trial)
Source: BMJ Open. 2026 Jun 25;16(6):e113058. doi: 10.1136/bmjopen-2025-113058 (PMC13311754; doi:10.1136/bmjopen-2025-113058)
Supplement: online supplemental file 1 [file bmjopen-16-6-s001.pdf]

Proefpersoneninformatie voor deelname aan medisch-wetenschappelijk onderzoek

Het effect van een dieet of medicatie op de levergezondheid bij mensen met overgewicht, diabetes, hoge bloedruk of hoog cholesterol

Officiële titel: Effect van mediterraan dieet gecombineerd met intermitterend vasten op leverfibrose in vergelijking met naltrexon/bupropion (Mysimba) bij mensen met cardiometabole risicofactoren (MEDFAST studie)

Inleiding

Geachte heer/mevrouw,

Met deze informatiebrief willen we u vragen of u wilt meedoen aan medisch-wetenschappelijk onderzoek. Meedoen is vrijwillig. U krijgt deze brief omdat u overgewicht heeft, met of zonder diabetes type 2, hoge bloeddruk of hoge cholesterolwaarden.

U leest hier om wat voor onderzoek het gaat, wat het voor u betekent, en wat de voordelen en nadelen zijn. Het is veel informatie. Wilt u de informatie doorlezen en beslissen of u wilt meedoen? Als u wilt meedoen, kunt u het formulier invullen dat u vindt in bijlage D.

Stel uw vragen

U kunt uw beslissing nemen met de informatie die u in deze informatiebrief vindt. Daarnaast raden we u aan om dit te doen:
- Stel vragen aan de onderzoeker die u deze informatie geeft.
- Praat met uw partner, familie of vrienden over dit onderzoek.
- Stel vragen aan de onafhankelijk deskundige, Dr. B. van Dalen. Voor contactgegevens zie bijlage A.
- Lees de informatie op www.rijksoverheid.nl/mensenonderzoek.

1. Algemene informatie
Het Franciscus heeft dit onderzoek opgezet. Hieronder noemen we het Franciscus steeds de ‘opdrachtgever’. Onderzoekers, dit kunnen ook artsen zijn, voeren het onderzoek uit in het Franciscus.

Deelnemers aan een medisch-wetenschappelijk onderzoek worden vaak proefpersonen genoemd. Zowel patiënten als mensen die gezond zijn, kunnen proefpersoon zijn. Wij verwachten dat 70 personen zullen deelnemen. De medisch-ethische toetsingscommissie MEC-U heeft dit onderzoek goedgekeurd. Innovative Health Initiative betaalt dit onderzoek.

2. Wat is het doel van het onderzoek?
In dit onderzoek bekijken we of een dieet en medicatie allebei leververlittekening kunnen verminderen bij mensen met overgewicht.

We willen deze twee methoden met elkaar vergelijken:
1. Een Mediterraan dieet waarbij deelnemers alleen mogen eten vanaf het ontbijt tot en met het avondeten (intermitterend vasten)
2. Een medicijn genaamd Mysimba®. Dit medicijn wordt vaker voorgeschreven bij mensen met overgewicht om af te vallen.

3. Wat is de achtergrond van het onderzoek?
In Nederland zijn er veel mensen met overgewicht, diabetes, hoge bloedruk en hoog cholesterol. Meer dan de helft van deze mensen heeft ook leververvetting. Leververvetting is een ophoping van vet in de lever en kan op den duur ontsteking geven, littekens vormen (fibrose) of zelfs afsterving van de lever geven (cirrose). Hierdoor kan de lever stoppen met werken. In de meest ernstige gevallen is een transplantatie van de lever nodig, maar dit komt gelukkig niet zo vaak voor. We weten dat afvallen kan helpen om leververvetting en leververlittekening te voorkomen en mogelijk zelfs te verminderen. Welke manier om af te vallen het beste is, dat weten we nog niet. Er zijn medicijnen om gewicht te verliezen, maar we weten ook dat gezonde voeding en leefstijl belangrijk is. In dit onderzoek zal de ene helft van de deelnemers het medicijn Mysimba gebruiken. Dit is een bestaand medicijn dat vaker bij mensen met overgewicht wordt voorgeschreven. De andere helft zal een Mediterraan dieet in combinatie met intermitterend vasten volgen. Intermitterend vasten betekent dat u ’s avonds niet meer mag eten tot de volgende ochtend. Hiermee onderzoeken we of zowel een dieet als medicatie helpt om leververvetting en leververlittekening bij mensen met overgewicht, diabetes, hoge bloedruk of hoog cholesterol te verminderen.

4. Hoe verloopt het onderzoek?
Hoelang duurt het onderzoek?
Doet u mee met het onderzoek? Dan duurt dat in totaal ongeveer zes maanden.

Stap 1: bent u geschikt om mee te doen?
We willen eerst weten of u geschikt bent om mee te doen. Daarom doet de onderzoeker een aantal onderzoeken:

- Onderzoek naar uw medische geschiedenis. Dit zal de onderzoeker telefonisch met u bespreken.
- Lichamelijk onderzoek. De onderzoeker bepaalt of u leverlittekening heeft. Dat doen we door een soort echo (Fibroscan) te maken. Hiervoor moet u naar het ziekenhuis komen. Als uw gewicht niet bekend is, zal de onderzoeker dit ook meten.

Stap 2: de behandeling
U gaat voor zes maanden een behandeling volgen onder begeleiding van ons onderzoeksteam.

Voor dit onderzoek maken we twee groepen:

- Groep 1. De mensen in deze groep krijgen een Mediterraan dieet en mogen maximaal 10 uur eten (bijvoorbeeld van 08:00 uur tot maximaal 18:00 uur).
- Groep 2. De mensen in deze groep krijgen het medicijn Mysimba.

Loting bepaalt welke behandeling u krijgt, dit heet 'randomisatie'. U mag dus niet zelf kiezen in welke groep u komt. Ook uw behandelend arts en de onderzoekers hebben geen invloed op de uitslag van de loting.

Dieetgroep: U volgt een energiebeperkt Mediterraan dieet. Dit dieet is al goed onderzocht bij mensen met overgewicht. Een energiebeperkt dieet betekent dat u iedere dag 500 calorieën minder eet dan dat u nodig heeft. Dit berekenen we met onder andere u gewicht, lengte en leeftijd. Met Mediterraan bedoelen we eten wat normaal gegeten wordt in gebieden rondom de Middellandse Zee, zoals groenten, fruit, noten, kip, vis en olijfolie. Daarnaast wordt u gevraagd maximaal 10 uur op een dag te eten, dit noemen we een 'tijdsrestrictie'. U mag van ongeveer 08.00 uur 's ochtends tot 18.00 uur 's avonds eten. U zal dus na het avondeten niks meer eten. Het is wél toegestaan om calorie-vrije dranken te drinken, denk dan aan water, light frisdranken en thee en koffie zonder suiker en melk. We verwachten dat u dit elke dag volhoudt voor 3 maanden, maar na die 3 maanden krijgt u de vrijheid om 1-2 keer per week de hele dag te mogen eten. Eerder onderzoek laat positieve resultaten zien op het gewicht en de bloedwaarden bij mensen die deze tijdrestrictie volgen. Er is alleen nog geen langdurig onderzoek gedaan. Tijdens de bezoeken op de poli in het ziekenhuis heeft u ook een gesprek met een diëtist waarbij het dieet wordt besproken.

Medicatiegroep: De eerste week gebruikt u Mysimba één keer per dag. Daarna gebruikt u het twee keer per dag gedurende zes maanden. De dosering zal in de eerste maand worden opgebouwd volgens een schema. Daarnaast heeft u tijdens de bezoeken op de poli in het ziekenhuis ook een gesprek met een diëtist waarbij voeding en beweging worden besproken. U wordt hierbij geadviseerd om voedingsadviezen volgens de Schijf van Vijf te volgen.

Voor beide groepen gaat de standaardbehandeling gewoon door. Dat betekent dat u de medicatie die u normaal gesproken gebruikt ook tijdens het onderzoek blijft gebruiken.

Stap 3: onderzoeken en metingen

Voor het onderzoek is het nodig dat u drie keer in zes maanden naar het ziekenhuis komt. Een bezoek duurt ongeveer 60 minuten. We doen de volgende onderzoeken bij ieder bezoek:

- Lichamelijk onderzoek. De onderzoeker meet uw gewicht, lengte en middelomtrek en meet uw bloeddruk.
- Echo van de lever (Fibroscan). De onderzoeker kan met deze echo de gezondheid van uw lever meten.
- Onderzoek van uw bloed. Daarvoor neemt de onderzoeker per keer zes buisjes bloed af. Alles bij elkaar nemen we 33 ml bloed per keer bij u af. Deze hoeveelheid geeft bij volwassenen geen problemen. Ter vergelijking: iemand die bloed geeft bij de bloedbank, geeft per keer 500 ml bloed. Met het bloedonderzoek testen we deze zaken:
    - Gaat het goed met uw bloedglucose?
    - Zien we effect op de gezondheid van uw lever?
    - Zien we effect op de gezondheid van uw hart en bloedvaten?
- U vult een vragenlijst in. De vragen gaan over algemene gegevens, leefstijl, medicatie, kwaliteit van leven en tevredenheid van de behandeling.
- U vult een voedingsdagboekje in waarin u aangeeft wat u drie dagen heeft gegeten en gedronken.
- U draagt in de eerste en laatste week van het onderzoek een klein apparaatje ter grootte van een euromunt. Met dit apparaatje meten we uw beweging. We bevestigen het apparaatje met een soort waterdichte pleister op uw bovenbeen. U kunt er kleding over dragen en uw dagelijkse dingen gewoon doen zoals douchen, slapen en sporten.

In bijlage C staat welke metingen we doen bij ieder bezoek.

Wat is er anders dan bij gewone zorg?

Normaal komt u misschien één keer per drie tot vier maanden bij uw arts, diabetesverpleegkundige of diëtist voor controle van uw bloedwaarden. De bezoeken die bij dit onderzoek horen, zijn in plaats van uw bezoeken bij de arts/diëtist. We proberen de studiebezoeken zo veel mogelijk te laten samenvallen met uw normale controlebezoeken.

5. Welke afspraken maken we met u?

We willen graag dat het onderzoek goed verloopt. Daarom maken we de volgende afspraken met u:

- U neemt het medicijn of volgt het dieet op de manier die de onderzoeker u heeft uitgelegd.
- U doet tijdens dit onderzoek niet mee aan een ander medisch-wetenschappelijk onderzoek.
- U komt naar iedere afspraak.
- U neemt contact op met de onderzoeker in deze situaties:
    - U wilt andere medicijnen gaan gebruiken. Ook als dit homeopathische middelen, natuurgeneesmiddelen, vitaminen of geneesmiddelen van de drogist zijn.
    - U wordt in een ziekenhuis opgenomen of behandeld.
    - U krijgt plotseling problemen met uw gezondheid.
    - U wilt niet meer meedoen met het onderzoek.
    - Uw telefoonnummer, adres of e-mailadres verandert.

Mag u zwanger worden tijdens het onderzoek?
Vrouwen die zwanger zijn of borstvoeding geven, kunnen niet meedoen aan dit onderzoek. Vrouwen mogen ook niet zwanger worden tijdens het onderzoek. Het is namelijk niet goed voor uw (ongeboren) kind als u flink af valt wanneer u zwanger bent of borstvoeding geeft. Ook mag Mysimba niet gebruikt worden tijdens de zwangerschap. De onderzoeker vertelt u hoe u het beste een zwangerschap voorkomt. Praat hierover met uw partner.

Toch zwanger?
Wordt u toch zwanger tijdens het onderzoek? Laat dit dan meteen weten aan de onderzoeker. U moet dan in overleg met de onderzoeker zo snel mogelijk stoppen met dit onderzoek.

6. Van welke bijwerkingen, nadelige effecten of ongemakken kunt u last krijgen?

Beide behandelingen kunnen leiden tot lagere bloedglucosewaarden. Daarom wordt u als u diabetes heeft gevraagd om uw bloedglucosewaarden op bepaalde tijden door te geven aan de diabetesverpleegkundige, zodat uw medicatie kan worden aangepast. Wanneer u toch een hypo (te lage bloedglucose) krijgt, volgt u het normale advies van uw diëtist/diabetesverpleegkundige op. Ook kan uw bloeddruk dalen als u afvalt. Wanneer u duizelig wordt, bijvoorbeeld bij het opstaan, of als u andere klachten heeft, neem dan contact op met het onderzoeksteam (zie bijlage A voor contactgegevens).

Voor het dieet: De tijd waarin u mag eten kan soms lastig zijn, met name in de avond. U moet namelijk vóór 18.00 uur gegeten hebben. Verder kunt u tijdens het dieet last krijgen van een verhoogd hongergevoel en/of hoofdpijn. Meestal is dit alleen zo in het begin van de diëten, u kunt contact opnemen met uw diëtist als u langer last heeft. Wanneer u klachten heeft, neem dan altijd contact op met het onderzoeksteam (zie bijlage A voor contactgegevens).

Voor de medicatie: Het gebruik van Mysimba kan bijwerkingen geven. De volgende bijwerkingen komen soms tot zelden voor (bij 10 tot 30 op de 100 mensen):
- Maagdarmklachten, zoals misselijk voelen, overgeven en verstopping.
- Hoofdpijn

De volgende bijwerkingen komen zelden voor (bij 1 tot 10 op de 100 mensen):
- Psychische klachten, zoals angst en moeite met slapen.
- (Draai)duizelig, slaperig, moe of sloom zijn
- Hartklachten, zoals hartkloppingen en versnelde hartslag. Zeer zelden pijn op de borst.
- Overmatig zweten, opvliegers, verhoogde bloeddruk.
- Droge mond en veranderde smaak
- Oorsuizen
- Trillende handen of voeten
- Kaalheid
- Huiduitslag en jeuk. Zeer zelden overgevoeligheid en galbulten.

De volgende bijwerkingen komen erg weinig voor (bij minder dan 1 op de 100 mensen):
- Evenwichtsstoornis en reisziekte
- Geheugenverlies
- Verhoogde leverenzymen of ontsteking van de galblaas.
- Moeite met het krijgen van een erectie (impotentie).

Doet u mee aan het onderzoek? Dan krijgt u de bijsluiter mee bij het middel.

Wat zijn de mogelijke ongemakken van metingen tijdens het onderzoek?
Bloedafnames kunnen pijn doen of een blauwe plek geven. Er wordt drie keer bloed bij u afgenomen tijdens de studie. Alles bij elkaar nemen we 99 ml bloed bij u af. Deze hoeveelheid geeft bij volwassenen geen problemen. Ter vergelijking: bij de bloedbank wordt 500 ml bloed per keer afgenomen.

Verder vragen we u om twee keer tijdens het onderzoek voor één week een klein apparaatje te dragen. Met dit apparaatje meten we uw beweging. We bevestigen het apparaat met een waterdichte pleister op uw bovenbeen. Dit kan wellicht wat hinderlijk zijn, maar u kunt er gewoon mee slapen, douchen en sporten.

7. Wat zijn de voordelen en de nadelen als u meedoet aan het onderzoek?

Meedoen aan het onderzoek kan voordelen en nadelen hebben. Hieronder zetten we ze op een rij. Denk hier goed over na, en praat erover met anderen.

Bij zowel het dieet als de medicatie kan het zijn dat u afvalt, gezonder wordt en minder medicatie nodig heeft, maar zeker is dat niet. Op elk moment tijdens dit onderzoek kunnen uw bloedwaarden en/of symptomen terugkomen of verslechteren.

Meedoen aan het onderzoek kan deze nadelen of gevolgen hebben:
- U kunt last krijgen van de bijwerkingen of nadelige effecten van het dieet of de medicatie.
- U kunt last hebben van de metingen tijdens het onderzoek.
- U moet zich houden aan de afspraken die horen bij het onderzoek. Wij vragen u om drie keer naar het Franciscus Gasthuis te komen voor een bezoek van 60 minuten.
- We vragen u om drie keer drie vragenlijsten in te vullen. De vragen kunnen mogelijk persoonlijk zijn. Het invullen kost ongeveer 20 minuten in totaal per keer.

Het is mogelijk dat er tijdens het onderzoek toevallig iets wordt ontdekt dat niet direct van belang is voor het onderzoek maar wel voor uw gezondheid. Zie ook paragraaf 10 over onverwachte ontdekkingen.

Wilt u niet meedoen?
U beslist zelf of u meedoet aan het onderzoek. Wilt u niet meedoen? Dan krijgt u de gewone zorg. Hieronder vallen uw gebruikelijke afspraken bij de diabetesverpleegkundige/huisarts en eventueel uw afspraken bij de diëtist. Uw arts kan u meer vertellen over de behandelingsmogelijkheden die er zijn en over de voor- en nadelen daarvan.

8. Wanneer stopt het onderzoek?

De onderzoeker laat het u weten als er nieuwe informatie over het onderzoek komt die belangrijk voor u is. De onderzoeker vraagt u daarna of u blijft meedoen.

In deze situaties stopt voor u het onderzoek:

- Alle onderzoeken volgens het schema zijn voorbij.
- Het einde van het hele onderzoek is bereikt. Dit is wanneer er in totaal 70 mensen hebben deelgenomen.
- U bent zwanger geworden.
- U wilt zelf stoppen met het onderzoek. Dat mag op ieder moment. Meld dit dan meteen bij de onderzoeker. U hoeft er niet bij te vertellen waarom u stopt. U krijgt dan weer de gewone zorg. De onderzoeker zal u nog wel uitnodigen voor een nacontrole.
- De onderzoeker vindt het beter voor u om te stoppen. De onderzoeker zal u nog wel uitnodigen voor een nacontrole.
- Een van de volgende instanties besluit dat het onderzoek moet stoppen:
  - Franciscus,
  - de overheid, of
  - de medisch-ethische commissie die het onderzoek beoordeelt.

Wat gebeurt er als u stopt met het onderzoek?
De onderzoekers gebruiken de gegevens en het lichaamsmateriaal (bloed) die tot het moment van stoppen zijn verzameld. Als u wilt, kan verzameld lichaamsmateriaal worden vernietigd. Geef dit door aan de onderzoeker.

Het hele onderzoek is afgelopen als alle deelnemers klaar zijn.

9. Wat gebeurt er na het onderzoek?

Kunt u de medicijnen blijven gebruiken?
De medicijnen die u heeft gebruikt bij het onderzoek, kunt u na het onderzoek blijven gebruiken als uw behandeld arts denkt dat u daar baat bij heeft en als u dat zelf ook wilt. Het kan wel zijn dat uw zorgverzekeraar de medicatie niet vergoed.

Krijgt u de resultaten van het onderzoek?
Ongeveer een jaar nadat het onderzoek is afgerond laat de onderzoeker u weten wat de belangrijkste uitkomsten zijn van het onderzoek.

10. Wat doen we met uw gegevens en lichaamsmateriaal?

Doet u mee met het onderzoek? Dan geeft u ook toestemming om uw gegevens en lichaamsmateriaal te verzamelen, gebruiken en bewaren.

Welke gegevens bewaren we?
We bewaren deze gegevens
- uw naam
- uw geslacht
- uw adres
- uw geboortedatum
- gegevens over uw gezondheid
- (medische) gegevens die we tijdens het onderzoek verzamelen

Welk lichaamsmateriaal bewaren we?
We verzamelen, gebruiken en bewaren buisjes bloed.

Waarom verzamelen, gebruiken en bewaren we uw gegevens en lichaamsmateriaal?
We verzamelen, gebruiken en bewaren uw gegevens en uw lichaamsmateriaal om de vragen van dit onderzoek te kunnen beantwoorden. En om de resultaten te kunnen publiceren.

Hoe beschermen we uw privacy?
Om uw privacy te beschermen geven wij uw gegevens en uw lichaamsmateriaal een code. Op al uw gegevens en lichaamsmateriaal zetten we alleen deze code. De code gebruiken we ook om het apparaatje in te stellen dat uw beweging meet. De sleutel van de code bewaren we op een beveiligde plek in het ziekenhuis. Als we uw gegevens en lichaamsmateriaal verwerken, gebruiken we steeds alleen die code. Ook in rapporten en publicaties over het onderzoek kan niemand terughalen dat het over u ging.

Wie kunnen uw gegevens zien?

Sommige personen kunnen wel uw naam en andere persoonlijke gegevens zonder code inzien. Dit kunnen gegevens zijn die speciaal voor dit onderzoek zijn verzameld, maar ook gegevens uit uw medisch dossier.
Dit zijn mensen die controleren of de onderzoekers het onderzoek goed en betrouwbaar uitvoeren. Deze personen kunnen bij uw gegevens komen:

- Leden van de commissie die de veiligheid van het onderzoek in de gaten houdt.
- Een controleur die door de opdrachtgever is ingehuurd.
- Nationale toezichthoudende autoriteiten.

Deze personen houden uw gegevens geheim. Voor inzage door deze personen vragen wij u toestemming te geven. De Inspectie Gezondheidszorg en Jeugd kan zonder uw toestemming uw gegevens inzien.

Hoelang bewaren we uw gegevens en lichaamsmateriaal?
We bewaren uw gegevens 25 jaar bij de opdrachtgever. Uw lichaamsmateriaal bewaren we in het ziekenhuis. Het wordt 25 jaar bewaard om daarop in de loop van dit onderzoek nog nieuwe bepalingen te kunnen doen die te maken hebben met dit onderzoek. Zodra dit niet meer nodig is, vernietigen we uw lichaamsmateriaal.

Mogen we uw gegevens en lichaamsmateriaal gebruiken voor ander onderzoek?
Uw verzamelde gegevens en uw (overgebleven) lichaamsmateriaal kunnen ook van belang zijn voor ander wetenschappelijk onderzoek op het gebied van overgewicht en leververlittekening. Daarvoor zullen uw gegevens en lichaamsmateriaal 25 jaar worden bewaard in het ziekenhuis. In het toestemmingformulier geeft u aan of u dit goed vindt. Geeft u geen toestemming? Dan kunt u nog steeds meedoen met dit onderzoek. U krijgt dezelfde zorg.

Wat gebeurt er bij onverwachte ontdekkingen?
Tijdens het onderzoek kunnen we toevallig iets vinden dat niet direct van belang is voor het onderzoek maar wel voor uw gezondheid. De onderzoeker neemt dan contact op met u en uw huisarts of specialist. U bespreekt dan met uw huisarts of specialist wat er moet gebeuren. De kosten hiervan vallen onder uw eigen zorgverzekering. U geeft met het formulier toestemming voor het informeren van uw huisarts of specialist.

Kunt u uw toestemming voor het gebruik van uw gegevens weer intrekken?
U kunt uw toestemming voor het gebruik van uw gegevens op ieder moment intrekken. Zeg dat dan tegen de onderzoeker. Maar let op: trekt u uw toestemming in, en hebben onderzoekers dan al gegevens verzameld voor het onderzoek? Dan mogen zij deze gegevens nog wel gebruiken. Voor uw lichaamsmateriaal geldt dat de onderzoekers dit vernietigen nadat u uw toestemming intrekt. Maar zijn er dan al metingen gedaan met uw lichaamsmateriaal? Dan mag de onderzoeker de resultaten daarvan blijven gebruiken.

Wilt u meer weten over uw privacy?
- Wilt u meer weten over uw rechten bij de verwerking van persoonsgegevens? Kijk dan op www.autoriteitpersoonsgegevens.nl.
- Heeft u vragen over uw rechten? Of heeft u een klacht over de verwerking van uw persoonsgegevens? Neem dan contact op met degene die verantwoordelijk is voor de verwerking van uw persoonsgegevens. Voor uw onderzoek is dat het Franciscus. Zie bijlage A voor contactgegevens.
- Als u klachten heeft over de verwerking van uw persoonsgegevens, raden we u aan om deze eerst te bespreken met het onderzoeksteam. U kunt ook naar de Functionaris Gegevensbescherming van het Franciscus gaan. Of u dient een klacht in bij de Autoriteit Persoonsgegevens.

Waar vindt u meer informatie over het onderzoek?
Op de volgende website vindt u meer informatie over het onderzoek: https://onderzoekmetmensen.nl/nl. Na het onderzoek kan de website een samenvatting van de resultaten van dit onderzoek tonen. U vindt het onderzoek door te zoeken op NL88048.100.24.

11. Krijgt u een vergoeding als u meedoet aan het onderzoek?
De extra testen en behandeling voor het onderzoek kosten u niets. U krijgt ook geen vergoeding als u meedoet aan dit onderzoek. Wel is er een reiskostenvergoeding beschikbaar voor de keren dat u naar

het Franciscus moet reizen. Dit geldt voor zowel benzinekosten als kosten voor het openbaar vervoer. Ook parkeerkosten kunnen worden vergoed.

12. Bent u verzekerd tijdens het onderzoek?

Voor iedereen die meedoet aan dit onderzoek is een verzekering afgesloten. De verzekering betaalt voor schade door het onderzoek. Maar niet voor alle schade. In **bijlage B** vindt u meer informatie over de verzekering en de uitzonderingen. Daar staat ook aan wie u schade kunt melden.

13. We informeren uw huisarts en behandelend specialist

De onderzoeker stuurt uw huisarts, diabetesverpleegkundige en/of behandelend specialist een e-mail om te laten weten dat u meedoet aan het onderzoek. Dit is voor uw eigen veiligheid.

14. Heeft u vragen?

Vragen over het onderzoek kunt u stellen aan het onderzoeksteam. Wilt u advies van iemand die er geen belang bij heeft? Ga dan naar de onafhankelijk deskundige dr. B. van Dalen, voor contactgegevens zie bijlage A. Hij weet veel over het onderzoek, maar werkt niet mee aan dit onderzoek.
Heeft u een klacht? Bespreek dit dan met de onderzoeker of de arts die u behandelt. Wilt u dit liever niet? Ga dan naar klachtencommissie van het Franciscus. In bijlage A staat waar u die kunt vinden.

15. Hoe geeft u toestemming voor het onderzoek?

U kunt eerst rustig nadenken over dit onderzoek. Daarna vertelt u de onderzoeker of u de informatie begrijpt en of u wel of niet wilt meedoen. Wilt u meedoen? Dan vult u het toestemmingsformulier in dat u bij deze informatiebrief vindt. U en de onderzoeker krijgen allebei een getekende versie van deze toestemmingsverklaring.

Dank voor uw tijd.

Met vriendelijke groet,
Namens het onderzoeksteam

Dr. Manuel Castro Cabezas, hoofdonderzoeker
Carmen Dietvorst, MSc., coördinerend onderzoeker

16. Bijlagen bij deze informatie

A. Contactgegevens
B. Informatie over de verzekering
C. Schema overzicht metingen
D. Toestemmingsformulier proefpersoon

Bijlage A: Contactgegevens

Hoofdonderzoeker:
Dr. Manuel Castro Cabezas
Internist-endocrinoloog, Vasculair specialist, afdeling Interne Geneeskunde (010) 4611234

Coördinerend onderzoeker:
Carmen Dietvorst, MSc.
Diëtist-onderzoeker, afdeling Interne Geneeskunde (010) 4611234

Onafhankelijk deskundige Franciscus:
Dr. B.M. van Dalen, cardioloog ******************

Klachten:
Franciscus
Telefonisch bereikbaar op werkdagen tussen 9.00-16.30 uur (010) 8934125
Digitaal via www.franciscus.nl/klacht

Functionaris voor de Gegevensbescherming:
Franciscus
FG@franciscus.nl

Voor meer informatie over uw rechten:
Indien u vragen heeft over de verwerking van uw persoonsgegevens kunt u zich per e-mail of post wenden tot de Functionaris Gegevensbescherming: FG@franciscus.nl.

Bijlage B: Informatie over de verzekering

Het Franciscus heeft een verzekering afgesloten voor iedereen die meedoet aan het onderzoek. De verzekering betaalt de schade die u heeft doordat u aan het onderzoek meedeed. Het gaat om schade die u krijgt tijdens het onderzoek, of binnen 4 jaar na het einde van uw deelname aan het onderzoek. U moet schade binnen 4 jaar melden bij de verzekeraar.

Heeft u schade door het onderzoek? Meld dit dan bij de verzekeraar. Bij schade kunt u ook contact opnemen met de cliëntvertrouwenspersoon van het Franciscus. Vermeld hierbij aan welke wetenschappelijke studie u deelneemt en waar deze verzekerd is.

Bereikbaarheid cliëntvertrouwenspersoon:
Franciscus, telefoonnummer (010) 893 4125

De verzekeraar van het onderzoek is:	
Naam:	Centramed
Adres:	Postbus 7374, 2701 AJ Zoetermeer
Telefoonnummer:	070 301 7070
E-mail:	schade@centramed.nl

De verzekering betaalt maximaal € 650.000 per persoon en € 5.000.000 voor het hele onderzoek en € 7.500.000 per jaar voor alle onderzoeken van dezelfde opdrachtgever.

Let op: de verzekering dekt de volgende schade **niet**:

- Schade door een risico waarover we u informatie hebben gegeven in deze brief. Maar dit geldt niet als het risico groter bleek te zijn dan we van tevoren dachten. Of als het risico heel onwaarschijnlijk was.
- Schade aan uw gezondheid die ook zou zijn ontstaan als u niet aan het onderzoek had meegedaan.
- Schade die ontstaat doordat u aanwijzingen of instructies niet of niet goed opvolgde.
- Schade aan de gezondheid van uw kinderen of kleinkinderen.
- Schade door een behandelmethode die al bestaat. Of door onderzoek naar een behandelmethode die al bestaat.

Deze bepalingen staan in het 'Besluit verplichte verzekering bij medisch-wetenschappelijk onderzoek met mensen 2015'. Dit besluit staat in de Wettenbank van de overheid (https://wetten.overheid.nl).

Bijlage C: Schema overzicht metingen

Afspraak	Wanneer	Wat
0	Voor de start	- Bezoek poli van ongeveer 30 minuten met onderzoeker<br>- Onderzoeker beantwoordt al uw vragen<br>- Bepalen of u leververlittekening heeft via een echo (Fibroscan)<br>- Tekenen toestemmingsformulier
1	Start	- Bezoek poli van ongeveer 60 minuten met onderzoeker<br>- Onderzoeker beantwoordt al uw vragen<br>- Navraag van uw voeding en/of medicatie<br>- Meten gewicht, middelomvang en lichaamssamenstelling (vetmassa/spiermassa)<br>- Bloed prikken en bloeddruk meten<br>- Vragenlijsten inleveren (thuis ingevuld)<br>- Een klein apparaatje dragen in de eerste week, zowel overdag als 's nachts
2	Na 2 weken	- Telefonische afspraak van ongeveer 15 minuten met onderzoeker<br>- Onderzoeker beantwoordt al uw vragen<br>- Navraag of het volgen van het dieet of het gebruik van de medicatie goed gaat
3	Na 3 maanden	- Bezoek poli van ongeveer 60 minuten met onderzoeker<br>- Onderzoeker beantwoordt al uw vragen<br>- Navraag van uw voeding en/of medicatie<br>- Meten gewicht, middelomvang en lichaamssamenstelling (vetmassa/spiermassa)<br>- Meten leververlittekening via een echo<br>- Bloed prikken en bloeddruk meten<br>- Vragenlijsten inleveren (thuis ingevuld)
4	Na 6 maanden	- Bezoek poli van ongeveer 60 minuten met onderzoeker<br>- Onderzoeker beantwoordt al uw vragen<br>- Navraag van uw voeding en/of medicatie<br>- Meten gewicht, middelomvang en lichaamssamenstelling (vetmassa/spiermassa)<br>- Meten leververlittekening via een echo<br>- Bloed prikken en bloeddruk meten<br>- Vragenlijsten inleveren (thuis ingevuld)<br>- Een klein apparaatje dragen in de laatste week, zowel overdag als 's nachts

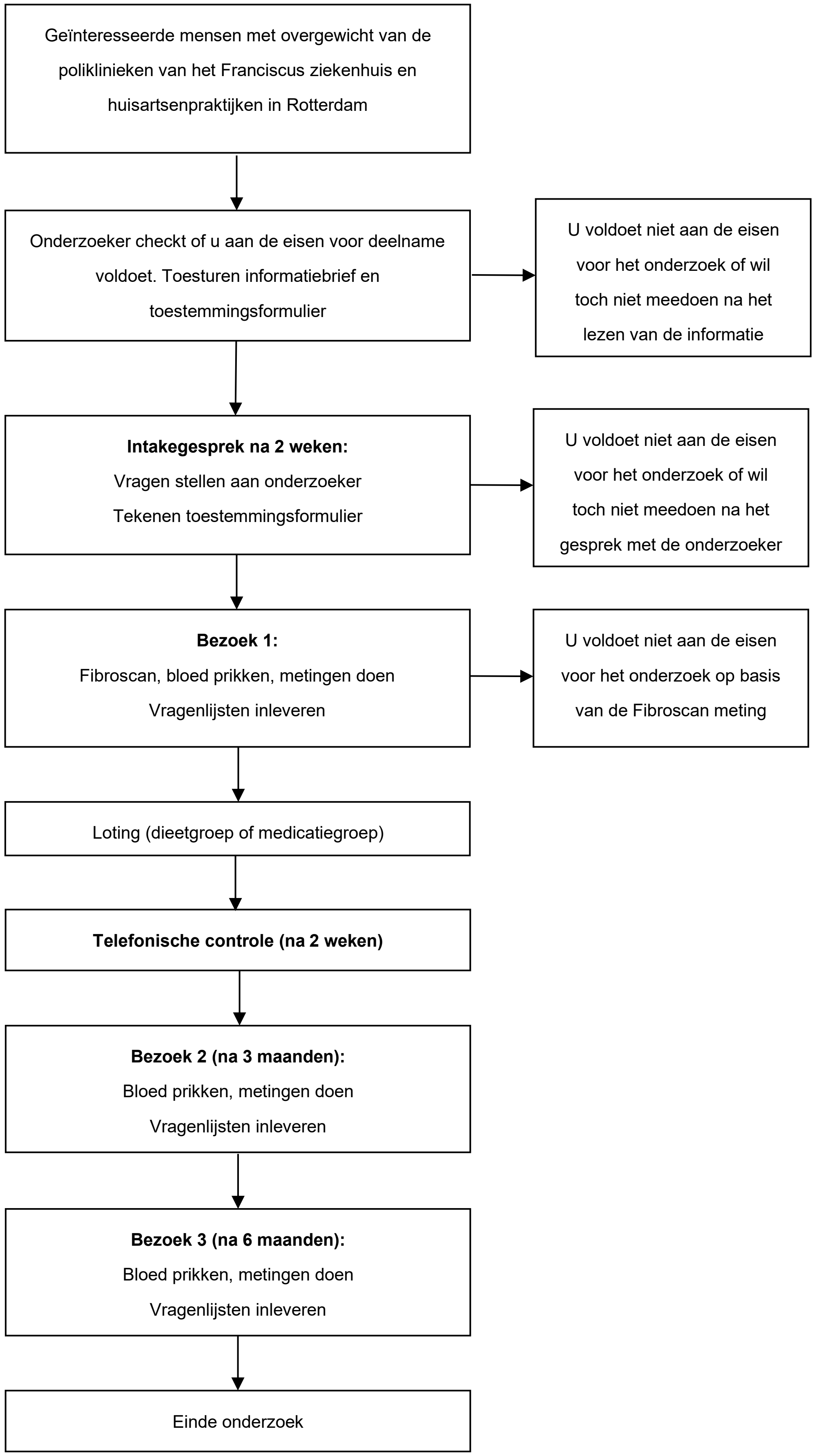
Geïnteresseerde mensen met overgewicht van de poliklinieken van het Franciscus ziekenhuis en huisartsenpraktijken in Rotterdam
Onderzoeker checkt of u aan de eisen voor deelname voldoet. Toesturen informatiebrief en toestemmingsformulier
U voldoet niet aan de eisen voor het onderzoek of wil toch niet meedoen na het lezen van de informatie
Intakegesprek na 2 weken:
Vragen stellen aan onderzoeker
Tekenen toestemmingsformulier
U voldoet niet aan de eisen voor het onderzoek of wil toch niet meedoen na het gesprek met de onderzoeker
Bezoek 1:
Fibroscan, bloed prikken, metingen doen
Vragenlijsten inleveren
U voldoet niet aan de eisen voor het onderzoek op basis van de Fibroscan meting
Loting (dieetgroep of medicatiegroep)
Telefonische controle (na 2 weken)
Bezoek 2 (na 3 maanden):
Bloed prikken, metingen doen
Vragenlijsten inleveren
Bezoek 3 (na 6 maanden):
Bloed prikken, metingen doen
Vragenlijsten inleveren
Einde onderzoek

Bijlage D: Toestemmingsformulier proefpersoon

Behorende bij:

Het effect van een dieet of medicatie om af te vallen bij mensen met overgewicht en leververlittekening

- Ik heb de informatiebrief gelezen. Ook kon ik vragen stellen. Mijn vragen zijn goed genoeg beantwoord. Ik had genoeg tijd om te beslissen of ik meedoe.
- Ik weet dat meedoen vrijwillig is. Ook weet ik dat ik op ieder moment kan beslissen om toch niet mee te doen met het onderzoek. Of om ermee te stoppen. Ik hoef dan niet te zeggen waarom ik wil stoppen.
- Ik geef de onderzoeker toestemming om mijn huisarts en specialist te laten weten dat ik meedoe aan dit onderzoek.
- Ik geef de onderzoeker toestemming om mijn huisarts of specialist informatie te geven over onverwachte bevindingen uit het onderzoek die van belang zijn voor mijn gezondheid.
- Ik geef de onderzoekers toestemming om mijn gegevens en/of lichaamsmateriaal te verzamelen en gebruiken. De onderzoekers doen dit alleen om de onderzoeksvraag van dit onderzoek te beantwoorden
- Ik weet dat voor de controle van het onderzoek sommige mensen al mijn gegevens kunnen inzien. Die mensen staan in deze informatiebrief. Ik geef deze mensen toestemming om mijn gegevens in te zien voor deze controle.
- Ik weet dat ik niet zwanger mag worden tijdens het onderzoek.
- De onderzoeker heeft met mij besproken hoe ik het beste voorkom dat ik zwanger word.

- Wilt u in de tabel hieronder ja of nee aankruisen?

Ik geef toestemming om mijn gegevens te bewaren om dit te gebruiken voor ander onderzoek, zoals in de informatiebrief staat.	Ja ☐	Nee☐
Ik geef toestemming om mijn (overgebleven) lichaamsmateriaal te bewaren om dit te gebruiken voor ander onderzoek, zoals in de informatiebrief staat. Het lichaamsmateriaal wordt daarvoor nog 25 jaar bewaard.	Ja ☐	Nee☐
Ik geef toestemming om mij eventueel na dit onderzoek te vragen of ik wil meedoen met een vervolgonderzoek.	Ja ☐	Nee☐

- Ik wil meedoen aan dit onderzoek.

Mijn naam is (proefpersoon): ………………………………..
Handtekening: ……………………… Datum : __ / __ / __

Ik verklaar dat ik deze proefpersoon volledig heb geïnformeerd over het genoemde onderzoek.

Wordt er tijdens het onderzoek informatie bekend die die de toestemming van de proefpersoon kan beïnvloeden? Dan laat ik dit op tijd weten aan deze proefpersoon.

Naam onderzoeker (of diens vertegenwoordiger):………………………………
Handtekening:……………………… Datum: __ / __ / __

De proefpersoon krijgt een volledige informatiebrief mee, samen met een getekende versie van het toestemmingsformulier.
