# Supplementary material for: Effect of an early time restricted eating Mediterranean diet compared to naltrexone/bupropion on liver fibrosis in people with cardiometabolic risk factors in a hospital outpatient clinic: study protocol for a randomised controlled trial (MEDFAST trial)
Source: BMJ Open. 2026 Jun 25;16(6):e113058. doi: 10.1136/bmjopen-2025-113058 (PMC13311754; doi:10.1136/bmjopen-2025-113058)
Supplement: Supplementary file 2 [file bmjopen-2025-113058.reviewer_comments.pdf]

PEER REVIEW HISTORY

BMJ Open publishes all reviews undertaken for accepted manuscripts. Reviewers are asked to complete a checklist review form and are provided with free text boxes to elaborate on their assessment. These free text comments are reproduced below.

ARTICLE DETAILS

Title (Provisional)

Effect of an early time restricted eating Mediterranean diet compared to naltrexone/bupropion on liver fibrosis in people with cardiometabolic risk factors in a hospital outpatient clinic: study protocol for a randomised controlled trial (MEDFAST trial)

Authors

Dietvorst, Carmen A.W.; Konings, Laura Anna; Saidi, Alina N.; Noordermeer, Tessa; Van Wenum, Martien; Peeters, Robin P.; Berk, Kirsten Anna; Castro Cabezas, Manuel

---

VERSION 1 - REVIEW

**Reviewer**	**1**
**Name**	**Zhao, Jingjie**
**Affiliation**	**Capital Medical University**
**Date**	**25-Dec-2025**
**COI**	**None**

This is a well-structured study protocol for a randomized controlled trial comparing the effects of early time-restricted eating combined with a Mediterranean diet (eTRE-MD) versus naltrexone/bupropion (N/B) on liver fibrosis in overweight/obese individuals with cardiometabolic risk factors. The research question is clinically significant, and the trial design is generally sound and adheres to SPIRIT guidelines. I recommend Minor Revision before acceptance, pending clarification and elaboration on the following points.

1.Precise Definition of the Primary Endpoint: The protocol states the primary endpoint is "liver fibrosis (kPa)" but does not specify the exact analytical metric—whether it is the absolute change from baseline, percent change, or endpoint value. While the "Statistical analysis" section mentions "change in liver fibrosis," to avoid ambiguity, it is recommended to clearly state in the "Outcomes" section (Page 10) that the primary endpoint is the absolute change in liver stiffness measurement (kPa) from baseline to 6 months.

2. Detailed Description of Dietary Intervention Implementation and Adherence Support:

Specific Dietary Protocol: The protocol mentions "based on the Mediterranean food pyramid" and "a daily calorie restriction of 500 kcal." To enhance operationalizability and replicability, please elaborate:

Will participants be provided with concrete examples, such as sample meal plans, recipes, or food exchange lists?

What is the specific content and frequency of dietitian guidance (beyond the visits at baseline, 3, and 6 months)? Will ongoing support be provided via telephone or digital tools?

How will the "daily 500 kcal deficit" be calculated and individualized for each participant? Will a standardized formula (e.g., Mifflin-St Jeor equation) be used?

Monitoring Time Restriction: How will adherence to the "8 AM to 6 PM" eating window be objectively monitored? Will participants be asked to use dietary logging apps or time-tracking tools?

3. Stronger Justification for the Choice of Comparator Drug (N/B):

The background section notes the regulatory approval of drugs like semaglutide and resmetirom for MASH/MASLD-related fibrosis. The choice of N/B—primarily a weight management agent—as the active comparator, rather than these agents with established liver indications, requires more substantial justification.

It is recommended to add a brief rationale in the "Introduction" or "Methods" section explaining the choice of N/B. Potential reasons could include: 1) The aim to compare a non-pharmacological (dietary) intervention against an oral, relatively lower-cost, widely used weight-loss medication; 2) Exploring the effect of weight loss per se (achieved via different modalities) on liver fibrosis; 3) Investigating an alternative strategy for patients unable or unwilling to use injectables (e.g., GLP-1 agonists) or novel targeted therapies (e.g., resmetirom). Clarifying this point is crucial for readers to understand the study's design logic and clinical positioning.

Reviewer 2

Name Kessoku, Takaomi

Affiliation Yokohama City University School of Medicine Graduate School of Medicine, Department of Gastroenterology and Hepatology

Date 29-Mar-2026

COI None

This study is a randomized controlled trial directly comparing a lifestyle intervention (eTRE combined with a Mediterranean diet) with pharmacological treatment (naltrexone/bupropion), which is novel and clinically relevant. In particular, the study addresses a real-world clinical dilemma in MASLD management (dietary intervention versus

medication) and uses liver stiffness as a clinically applicable endpoint. However, there are several important methodological concerns and ambiguities in the protocol that should be addressed. Therefore, major revision is required.

Major Comments

1, Definition of MASLD population

It is unclear whether this study appropriately targets patients with MASLD. The inclusion criteria rely primarily on liver stiffness measurements without sufficient evidence of hepatic steatosis (e.g., imaging such as ultrasound or CAP). Since MASLD requires the presence of steatosis, the current definition may be insufficient and should be clarified.

2, Validity of LSM as a fibrosis endpoint

Liver stiffness measurement (LSM) does not directly reflect liver fibrosis and can be influenced by inflammation, congestion, or metabolic changes. Therefore, the manuscript should avoid interpreting LSM as a direct surrogate for fibrosis and revise the wording accordingly.

3, Clinical rationale for comparing diet vs pharmacotherapy

The clinical rationale for directly comparing a dietary intervention with pharmacological treatment remains unclear. The authors should more clearly justify why these two fundamentally different treatment strategies are being compared and how this comparison informs clinical decision-making.

Medication adherence in the N/B group

4, The protocol lacks sufficient detail regarding how adherence to naltrexone/bupropion will be assessed and ensured. More detailed information on compliance monitoring and its impact on the analysis should be provided.

Minor Comment

1, Definition of alcohol intake

The definition of alcohol consumption should be standardized and reported in grams per day (g/day) rather than number of drinks to improve clarity and international comparability.

VERSION 1 - AUTHOR RESPONSE

Reviewer: 1

This is a well-structured study protocol for a randomized controlled trial comparing the effects of early time-restricted eating combined with a Mediterranean diet (eTRE-MD) versus naltrexone/bupropion (N/B) on liver fibrosis in overweight/obese individuals with cardiometabolic risk factors. The research question is clinically significant, and the trial design is generally sound and adheres to SPIRIT guidelines. I recommend Minor Revision before acceptance, pending clarification and elaboration on the following points.

1.Precise Definition of the Primary Endpoint: The protocol states the primary endpoint is "liver fibrosis (kPa)" but does not specify the exact analytical metric—whether it is the absolute change from baseline, percent change, or endpoint value. While the "Statistical analysis" section mentions "change in liver fibrosis," to avoid ambiguity, it is recommended to clearly state in the "Outcomes" section (Page 10) that the primary endpoint is the absolute change in liver stiffness measurement (kPa) from baseline to 6 months.

Thank you for this valid point. We have changed 'liver fibrosis' to 'absolute change in liver fibrosis' (page 10, line 280) and 'liver steatosis' to 'absolute change in liver steatosis' (page 10, line 285) in the Outcomes section. In the 'Statistical analysis' section, we have added the word 'absolute' to 'change in liver fibrosis' (page 14, line 396) for more clarity.

2. Detailed Description of Dietary Intervention Implementation and Adherence Support: Specific Dietary Protocol: The protocol mentions "based on the Mediterranean food pyramid" and "a daily calorie restriction of 500 kcal." To enhance operationalizability and replicability, please elaborate:

- Will participants be provided with concrete examples, such as sample meal plans, recipes, or food exchange lists?
  We added the following text on page 9, lines 248-250: "At baseline, participants receive a detailed explanation of the dietary protocol, two sample daily meal plans, recipes, and a food-exchange list".

- What is the specific content and frequency of dietitian guidance (beyond the visits at baseline, 3, and 6 months)? Will ongoing support be provided via telephone or digital tools?
  To ensure that the diet group remains comparable to the medication group, participants in the diet group will not receive additional scheduled appointments with the dietitian. They may, however, contact the dietitian on their own initiative if they have any questions. We have clarified this by adding the following text on page 9, lines 256-258: "Throughout the intervention period, participants may also contact the study dietitian by telephone for ongoing support".

- How will the "daily 500 kcal deficit" be calculated and individualized for each participant? Will a standardized formula (e.g., Mifflin-St Jeor equation) be used?
  We will use either the WHO equation or the Harris-Benedict equation, depending on the participant's BMI. We have revised this on page 9, lines 244-246 to: "calculated using the WHO equation for participants with a BMI ≤ 30 and the Harris-Benedict equation for those with a BMI > 30".

- Monitoring Time Restriction: How will adherence to the "8 AM to 6 PM" eating window be objectively monitored? Will participants be asked to use dietary logging apps or time-tracking tools?
  We have provided a more detailed explanation of adherence by adding the following text on page 9, lines 250-254: "Dietary adherence is assessed at the 3-month and 6-month follow up visits using food diaries in which participants record all foods consumed and the corresponding times. The eating window is additionally evaluated

at each visit through a questionnaire assessing the average latest eating time and the number of days per week participants adhere to the fasting regimen".

We thank the reviewers for these valuable suggestions. We agree that additional detail is needed to improve the operationalization and reproducibility of the dietary protocol. We have therefore expanded the description of the intervention in the Methods section, as mentioned above.

3. Stronger Justification for the Choice of Comparator Drug (N/B):
The background section notes the regulatory approval of drugs like semaglutide and resmetirom for MASH/MASLD-related fibrosis. The choice of N/B—primarily a weight management agent—as the active comparator, rather than these agents with established liver indications, requires more substantial justification.
It is recommended to add a brief rationale in the "Introduction" or "Methods" section explaining the choice of N/B. Potential reasons could include: 1) The aim to compare a non-pharmacological (dietary) intervention against an oral, relatively lower-cost, widely used weight-loss medication; 2) Exploring the effect of weight loss per se (achieved via different modalities) on liver fibrosis; 3) Investigating an alternative strategy for patients unable or unwilling to use injectables (e.g., GLP-1 agonists) or novel targeted therapies (e.g., resmetirom). Clarifying this point is crucial for readers to understand the study's design logic and clinical positioning.

We agree that clarification of the comparator choice is important. We added an explanation in the Introduction section that naltrexone/bupropion was chosen because the aim of this study is to isolate the effect of weight loss on liver fibrosis, on page 5, lines 132-137: "Importantly, agents such as semaglutide and resmetirom have direct cardiometabolic and liver specific effects beyond weight reduction, making it difficult to attribute changes in liver fibrosis to weight loss alone. In contrast, N/B induces weight loss without known hepatic benefits, making it an appropriate comparator for evaluating weight loss driven effects on MASLD, which is the aim of the current study".

Reviewer: 2
This study is a randomized controlled trial directly comparing a lifestyle intervention (eTRE combined with a Mediterranean diet) with pharmacological treatment (naltrexone/bupropion), which is novel and clinically relevant. In particular, the study addresses a real-world clinical dilemma in MASLD management (dietary intervention versus medication) and uses liver stiffness as a clinically applicable endpoint. However, there are several important methodological concerns and ambiguities in the protocol that should be addressed. Therefore, major revision is required.

Major Comments
1, Definition of MASLD population
It is unclear whether this study appropriately targets patients with MASLD. The inclusion criteria rely primarily on liver stiffness measurements without sufficient evidence of hepatic steatosis (e.g., imaging such as ultrasound or CAP). Since MASLD requires the presence of steatosis, the current definition may be insufficient and should be clarified.

We thank the reviewer for this comment. MASLD indeed requires evidence of hepatic steatosis. Our primary inclusion criterion focuses on liver stiffness because fibrosis is the strongest prognostic factor for adverse clinical outcomes in MASLD. We intentionally target a more advanced MASLD population, as there is limited evidence on the effects of dietary interventions in individuals with established fibrosis; an observation also supported by a systematic review being prepared by our group at present. We added an explanation for the choice at the Objectives section on page 6, lines 153-158: "Given that liver fibrosis is the strongest prognostic marker for adverse outcomes in MASLD, liver stiffness is the primary objective. This study intentionally targets a more advanced MASLD population, as there is limited evidence on the effects of dietary interventions in individuals with established fibrosis. Steatosis will also be analysed to enable comprehensive phenotyping and to examine its relationship with changes in liver stiffness and metabolic outcomes.".

2, Validity of LSM as a fibrosis endpoint
Liver stiffness measurement (LSM) does not directly reflect liver fibrosis and can be influenced by inflammation, congestion, or metabolic changes. Therefore, the manuscript should avoid interpreting LSM as a direct surrogate for fibrosis and revise the wording accordingly.

We agree that liver stiffness measurement (LSM) does not directly quantify fibrosis and can be influenced by factors such as inflammation or congestion. At the same time, liver biopsy is not a feasible or appropriate method for repeated assessment in this study. LSM is widely accepted as a non-invasive tool for evaluating liver fibrosis severity and monitoring disease progression, and has been acknowledged by the FDA as a "reasonably likely surrogate endpoint" for clinical trials in MASH. According to the FDA, LSM correlates with fibrosis severity, may predict clinical outcomes, and provides a safer and more accessible alternative to biopsy (https://www.fda.gov/drugs/drug-safety-and-availability/fda-accepts-proposal-reasonably-likely-surrogate-endpoint-mash-all-cause-mortality-or-liver-related). To clarify this, we have added in the manuscript "measured as liver stiffness by transient elastography" after "liver fibrosis" on page 2, line 44 and 49; page 6, line 152; page 7, line 199-200.

3, Clinical rationale for comparing diet vs pharmacotherapy
The clinical rationale for directly comparing a dietary intervention with pharmacological treatment remains unclear. The authors should more clearly justify why these two fundamentally different treatment strategies are being compared and how this comparison informs clinical decision-making.

The main aim is to help people lose weight and thereby improve their liver health. Current guidelines recommend lifestyle modification as the first-line treatment, but several pharmacological options are now available to support weight loss. Because some patients respond better to lifestyle interventions while others respond better to medication, directly comparing these two primary treatment strategies helps determine whether their effectiveness differs. We have explained this a little more clearly in the introduction on page 5, lines 141-144: "Because both lifestyle modification and pharmacological therapy are considered primary treatment strategies – and individual patients may respond differently to each – direct comparison of these approaches may help clarify their relative effectiveness and inform clinical decision-making."

4, Medication adherence in the N/B group

The protocol lacks sufficient detail regarding how adherence to naltrexone/bupropion will be assessed and ensured. More detailed information on compliance monitoring and its impact on the analysis should be provided.

We thank the reviewer for this comment. We have expanded the Methods section to provide more detail on adherence monitoring:
- Page 10, lines 269-273: "Compliance with N/B intake is monitored by pill counting at the three- and six-month visits. Participants are asked to bring all medicine packaging to each visit, and the number of remaining tablets is recorded by the researcher. Adherence is defined a priori as taking at least two tablets per day, with dose adjustments permitted based on individual tolerance".
- Page 14, lines 389-391: "These adherence data will be used to classify participants for the per protocol analysis and to explore the impact of medication compliance on treatment outcomes".

Minor Comment

1, Definition of alcohol intake

The definition of alcohol consumption should be standardized and reported in grams per day (g/day) rather than number of drinks to improve clarity and international comparability.

We have amended this in the list of exclusion criteria to "Known or suspected excessive alcohol consumption (>30 grams/day for males or >20 grams/day for females. One drink is equivalent to 10 grams of alcohol)" on page 9, lines 226-227.
